# Special Issue on Psychology of Uncertainty and Vulnerabilities: COVID-19 Pandemic Related Crisis

**DOI:** 10.1007/s12646-021-00623-w

**Published:** 2021-10-05

**Authors:** Tholene Sodi, Buxin Han, Purnima Singh

**Affiliations:** 1 Department of Psychology, University of Limpopo, Sovenga, South Africa; 2 Institute of Psychology, Chinese Academy of Sciences (CAS), Beijing, 100101 People’s Republic of China; 3 Department of Psychology, University of the CAS, Beijing, 100049 People’s Republic of China; 4 Department of Humanities and Social Sciences, Indian Institute of Technology Delhi, Hauz Khas, New Delhi, 110 016 India

What happens when what is more or less predictable in our lives becomes uncertain? Simple things like going out for work, visiting friends and relatives, or even grocery shopping have been uncertain for most of us in all regions of the world for almost two years, since the outbreak of the COVID-19 pandemic. Many such uncertainties about our present as well as future, unfolded during the various waves of COVID-19. Throughout the world, most people experienced uncertainties about health, economic, social, and personal lives. This has impacted our health, wellbeing, productivity, and posed many challenges for all of us. As Anderson et al. (2019) point out, uncertainty is a mental state, a subjective or cognitive experience that many people experience as aversive (Carleton, 2016). Individuals and communities are all facing what has been called the “new normal”, with significant changes in our lifestyles and the way we work and function. Like it is for many social issues, it is true that marginalized and vulnerable groups all over the world have been hardest hit by the pandemic. In light of this ravaging health emergency, Psychological Studies decided to bring out this special issue on *“Psychology of Uncertainty and Vulnerabilities: Pandemic Related Crisis*.”

We chose “Psychology of uncertainty and vulnerabilities” as the focus, because there is a close linkage between uncertainty and vulnerabilities as evidenced in literature on environmental hazards (Berkes, 2007). Understanding uncertainty helps in reducing vulnerability, as it may allow us to plan things better, mitigate some of the risks, and adapt more successfully. Many societies and communities have learnt to live with a myriad of uncertainties. Most people living in developing societies and especially global south, are adept with coping with unpredictability and uncertainties. People develop their own protocols of behaviour to cope with these uncertainties, and often such protocols are shared and become part of community and institutional memory. “Learning to live with uncertainty requires building a memory of past events, abandoning the notion of stability, expecting the unexpected, and increasing the capability to learn from crisis.” (Berkes, 2007; p.288). This facilitates adapting to the challenges of uncertainty.

Robinson (2020) posited that uncertainty can cause tremendous anxiety. Human beings prefer psychological stability and deride uncertainty. When our certainty is questioned, stress responses set in, instantly arousing some discomfort (Robinson, 2020). In case of COVID- 19, the uncertainty, the uncontrollability, the sudden changes, and the fear of the unknown caused anxiety, fear, stress etc. However, there is a positive side of uncertainty too. It may facilitate out of box thinking and innovation, as demonstrated by various vaccines and testing facilities, developed in a short span the world over. Han et al., (2011) suggest that there are three sources of uncertainty, which are salient in trying to explain responses to COVID-19. The first source is the probability or risk of the coronavirus, which arises from indefinite nature of the disease and the future health scenario worldwide. Secondly, the very nature of the situation is ambiguous (Ellsberg, 1961), due to inadequate information about probability of occurrence of the disease and its severity. The third source suggests that complexity results from difficulty in comprehending available information about disease, as research is in its initial stages, and leading to the use of ad hoc measures. We have also seen that uncertainty is a breeding ground for conspiracy theories that may result in negative emotional states. In the case of the COVID-19 pandemic, fear of the unknown (Carleton, 2016) also has influenced our negative feelings.

Thus, the linkages between uncertainty and vulnerability become more daunting, and consequently the title Psychology of Uncertainty and Vulnerabilities was preferred. In fact, the uncertainties arising during COVID-19 have made a majority of the population vulnerable, irrespective of class, region, race, and other stratifications. The pandemic has had such a huge impact, affecting all major regions of the world, all caught by unpreparedness. The onset, the aetiology, and our responses were all unclear. Everyone was overawed with the magnitude of this extraordinary health emergency, resulting in an acute sense of helplessness and fear. But those on the periphery were the worst hit. These represent the vulnerable sections of the society. In the context of natural hazards, Varnes (1984) posits that vulnerability is the potential damage that can be expected, depending on the characteristics, which have an element of risk. In case of COVID-19, vulnerability would mean the extent to which people are at risk and susceptible to the impact of the disease. Levels of uncertainty can increase vulnerability. Daily wagers, slum dwellers, poor, marginalized sections of the society are generally faced with many kinds of uncertainties in their lives, which may also make them vulnerable to negative impact of the pandemic. As Adger (2006) explains, vulnerability is the state of susceptibility to harm from exposure to stresses, as well as inability to adapt. Thus, the notions of adaptation and risk are central to the understanding of vulnerability. The degree of vulnerability depends on the characteristics of the risk, in this case, the pandemic and the individual’s or society’s ability to respond to the risk and to adapt to it. The marginalized tend to be vulnerable, because of their restricted access to resources, and limited ability to respond to risk and adapt to the risk. These sections of the society become more vulnerable to present and future loss, and this vulnerability is caused by uncertainty.

This Special Issue of Psychological Studies brings together different theoretical and empirical perspectives by scholars, whose contributions are aimed at improving our understanding of the psychology of uncertainty and vulnerabilities associated with the COVID-19 pandemic. Drawn from different parts of the worlds, the contributions focus mainly on four broad themes: meanings attached to the COVID-19 pandemic; psychological reactions and factors that might mitigate against the impact of the pandemic; short- and long-term psychological impact of the pandemic; and, individual and collective responses to deal with the pandemic and its psychological impact. The twelve papers that we briefly introduce below give some insights into the above-mentioned four themes and provide readers with an opportunity to further explore the psychology of uncertainty and vulnerabilities.

## Meanings Attached to the COVID-19 Pandemic

With regard to the first theme, Kučera reports on the results of a study that focused on the psychological-linguistic analysis of utterances by 2522 respondents, on the COVID-19 situation in the Czech Republic. The data was collected within a bigger research project that required the respondents to answer open questions related to their perception and experience of the Covid-19 situation (i.e., utterances), and their emotional states in recent days. For this article that we have included in the Special Issue, Kučera performed a series of psychological-linguistic analyses that relate to the utterances of the respondents to two posited questions: (a). What does the current situation mean to you; and (b). Has it changed your life in any way? The second question was accompanied by follow up questions such as: (a). If the current situation has changed your life, how so? (b). How do you currently experience the situation? (c). What do you consider the worst? (d). What helps you? One of the key findings of the study is that, the most distinctive utterances are connected to negative emotions, and most frequently relate to social environment, anxiety, and inhibition. The study gives valuable insights into how people use words, when facing an unexpected and emotionally disturbing situation, such as COVID-19 pandemic.

While the first article focuses on the psycho-linguistic meanings embedded in utterances by people in the Czech Republic, the second one—a theoretical contribution by Ehigie, seeks to highlight the challenges associated with the adoption of social distancing, as a way to prevent the transmission of COVID-19 in an African setting. Whilst social distancing is encouraged as one of the most common non-pharmaceutical interventions or measures to prevent the spread of a contagious disease, it is important to acknowledge that culture plays a critical role in the adoption and sustainable implementation of such public health measures. In his paper, Ehigie suggests that the traditional African communal or collective pattern of living has the potential to pose a challenge, when social distancing is to be implemented as an intervention to prevent the spread of COVID-19 in some African communities. The implication here is that, there is a need for public health officials to take into account the socio-cultural realities of the target communities, when implementing interventions.

## Psychological Reactions and the Associated Mitigating Factors

As previously stated elsewhere, uncertainty results in a stress response (Robinson, 2020) that will trigger further psychological reactions. Building on this theme, Da Silveira Riter and her colleagues investigated common mental disorders (CMD) symptoms in Brazilian parents during the COVID-19 pandemic. The authors found that perceived stress was the most related variable to parents’ symptoms of common mental disorders, showing a positive association. The results, also suggested that lower family income may increase symptoms of common mental disorders in Brazilian parents. With Brazil considered as one of the 10 countries with high social inequality, this finding is certainly worrisome. In concluding their paper, the authors recommend that interventions, targeting parental competence and stress reduction should be considered, in order to address the mental health impacts of COVID-19 pandemic.

In an online survey that sought to study regulatory resources and emotional states in overcoming associated with the lockdown in the Russian Federation, Morosanova, Bondareko, and Kondratyuk found that conscious self-regulation in conditions of self-imposed self-isolation is a useful resource for successful self-organization, and coping with anxiety associated with uncertainty, such as the one occasioned by COVID-19. The authors conclude by suggesting that conscious self-regulation serves as a great resource for self-organization during a lockdown; this has the potential to provide flexible adjustment during moments of uncertainty and when faced with new challenges. Additionally, it was found that an optimistic attitude does positively affect the conscious self-regulation of human activity.

## Short- and Long-Term Psychological Impact of the Pandemic

Building on the above papers on the psychological reactions to the pandemic and the associated possible mediating factors, Zinchenko et al., investigated the immediate impact of COVID-19 on the mental health of university in Russia. The study found that there was an increase in the levels of depression, anxiety, and depression between the beginning of March, 2020, when the first case of COVID-19 was officially confirmed in Moscow, and the time when the restrictive measures were adopted in Russia. Also, interestingly, the study found that there was an increase in the levels of depression, anxiety, and stress in young men, when compared to young women during the same period. Among others, the authors concluded that it is important to take into account the dynamics of students’ mental state and gender differences, when studying the impact of the pandemic. Singh and Quraishi also looked at the psychological challenges faced by students in India during the pandemic. They found that students faced significant challenges during their studies and that, this state of affairs tended to increase their mental stress.

While the article by Zinchenko et al. in Russia and the one by Singh and Quraishi in India, focused on the psychological impact of the pandemic on students, other studies done elsewhere, focused on the adult population. Putinas-Neugebauer and Roland-Lévy assessed the mental health consequences of the German population, using a cross-sectional online survey. The results revealed a prevalence of depressive symptoms, psychological discomfort, threat perception, generalized anxiety disorder, and sleep disturbances that were associated with the pandemic. In Spain, Saiz et al. investigated the impact of COVID-19, and the subsequent stay-at-home orders on the Spanish population’s sense of belonging at three moments in time. These were at the beginning of the lockdown, after a month of the lockdown, and with what they described as the return to the “new normal”. The authors found that a sense of belonging increased significantly during the lockdown and confinement, dropping dramatically with the start of the return to the “new normality” process. In conclusion, Saiz et al. call for actions to increase in our sense of belonging beyond the crisis caused by the COVID-19 pandemic.

An illustration of the impact of the pandemic on employees working in private organizations is provided by Varshney in a qualitative study that was conducted with 22 middle-level Indian employees. The study found that employees experienced psychological stress, social disconnectedness, and a sense of loneliness during the lockdown period in India. The author implores management in the private sector to be cognizant of the mental health issues of employees, and further suggests the need to alleviate employees’ professional and psychological challenges. The need to consider mental health issues as a result of the COVID-19 pandemic, also applies at the domestic level. In their paper, reflecting on the impact of COVID-19 in South Africa, Nduna and Tshona suggest that the implementation of lockdown restrictions has increased the risk of domestic violence for some women and girls. The authors argue that this is because the lockdown restrictions curtailed movements, thus increasing the risk of domestic violence. They suggest that the reduction of all forms of violence can help avert the risk of domestic violence that some women face in South Africa.

## Individual and Collective Responses to Deal with the Pandemic

In this final section of our editorial piece, two articles provide some insights into individual and collective responses to deal with the COVID-19 pandemic. Mathew et al. sought to examine positive mental health of the Indian population, focusing particularly on the Generations Y and Z that tend to form the majority of the working population. Their findings, suggest that positive mental health is not dependent on gender or age. Significantly, the authors developed a model on Positive Mental Health, based on the dimensions of the Positive Mental Health Instrument. The last paper in this Special Issue, authored by Jia and An, gives a comprehensive description of the actions that the Registration Work Committee of the Chinese Psychological Society took in response to the outbreak of the COVID-19 pandemic. Among others, the Work Committee developed and introduced guidelines for psychological support to be delivered remotely, and provided specific types of training, such as psychological first aid to psychological assistance professionals. The authors conclude by suggesting that the training and supervision have played an important role in mitigating the harsh impact of COVID-19 in China.

## Concluding Remarks

The 12 articles that we have included in this Special Issue, give us a glimpse of the psychology of uncertainty and vulnerabilities associated with the COVID-19 pandemic. The contributions focus mainly on four broad themes, namely: meanings attached to COVID-19 pandemic; psychological reactions and factors that might help to mitigate against the impact of the pandemic; short- and long-term psychological impact of the pandemic; and, individual and collective responses to deal with the pandemic and its psychological impact. These papers that we have put together do not, by any means, provide an exhaustive coverage of what is clearly a global pandemic of catastrophic proportions. We are aware that there are many other studies that have been published on the psychological factors, associated with coronavirus and related pandemics that have occurred in the past. We hope that future research will benefit from the contributions assembled in this Special Issue, and that these offerings will generate interest for more studies on the psychology of uncertainty and vulnerabilities.

## Data Availability

Not applicable.
